# Time to end parachute science

**DOI:** 10.1371/journal.pmed.1004099

**Published:** 2022-09-06

**Authors:** Beryne Odeny, Raffaella Bosurgi

**Affiliations:** PLOS Medicine, San Francisco, California, United States of America

Colonial science, also known as parachute or parasitic science, is an extractive practice whereby researchers—typically from highly resourced countries—do research and extract data and samples from non-native regions or populations, typically low resource settings or countries, [1] without appropriately acknowledging the importance of the local infrastructure and expertise. In so doing, foreign researchers fail to establish long term, equitable collaborations with local partners [2].

The era in which we are living is profoundly impacted by the effects of globalization, inequity, poverty, conflicts, climate change, biodiversity loss, and pandemics. Many of the solutions to these global health challenges come from sustainable and socially responsible behavior from societies; often, robust scientific evidence comes from collaborations among key opinion leaders, scientists, funders, policy makers, and local and international stakeholders across different countries [3]. For research to be sustainable and equitable, it should be founded on inclusive scientific liaison between varied collaborators—for example, between high income countries (HICs) and low- and middle-income countries (LMICs), and early-career researchers and established scientists. Unfortunately, inclusivity and equity are not the reality in most global research [2].

An indicator of this imbalance is the striking disparity in the quantity of publications by researchers in HICs compared to other regions [4]. This disparity has been reported as far back as 2 decades ago—one study illustrated that only 6.5% of research articles in general medical journals had a coauthor from the country where the study population lived [5]. A 2016 publication showed that less than 50% of infectious disease publications from Africa had an African first or last author [6]. More recently, a bibliometric study demonstrated increasing numbers in first and last authorship among sub-Saharan African (SSA)-affiliated authors in publications about SSA [4]. In geoscience, only 30% of articles from Africa had an African author [7]. In the field of coral reef biology, 40% of publications that contained fieldwork conducted in Indonesia or in the Philippines did not specify which nation the field research had been conducted in; the respective figure for Australia was just 22% [1]. While the engagement of local researchers is steadily increasing in fields like global health, scholarly inequities continue to be sustained through authorship hierarchies in which local authors are by default assigned middle-author positions, i.e., neither first nor last author positions [6,8]. Further, collaborative authorship models commonly involve assignment of robust primary outcomes papers—the cream of the research—to researchers from HICs, while secondary papers are allocated to local scientists.

These hierarchies in the author by-line perpetuate “cross-Atlantic” academic imperialism through systematic exclusion of less-advantaged researchers from prominent authorship positions—positions that are correlated with access to funding, future publications, promotions, and academic tenure [4,9]. More importantly, because scientists in HICs have majority access to funding, they tend to dictate the research agenda, thus the disease priorities of the countries hosting field research are not prioritized [10,11]. This is an ethical concern and should be a strong motivator to support local scientists, who are most affected by the implications of the science, to lead the conceptualization of research and writing of articles specific to their contexts [10,11].

From the societal angle, lack of equitable inclusion of local scientists has repercussions on community engagement, trust, and robustness of research, with profound effects on the outcome of the research, its reproducibility, and implementation [12]. For example, parachute science limits the effectiveness of responses to outbreaks of infectious diseases, such as those associated with the Ebola and Zika epidemics, in which lack of data sharing by foreign researchers undermined host countries’ capacity to prevent and prepare for outbreaks [13,14].

## What strategies need to be in place to curb parachute science?

Establishing equitable research collaborations is an invaluable strategy in equalizing global health research. First, there needs to be grateful recognition that the ability to conduct foreign research is an opportunity and not a right; it must involve local stakeholders in defining the research priorities, conceptualizing, and designing research before seeking funding for the project [3,9]. Second is to build the capacity of local scientists to analyze data, write publications, and empower them to play leading roles in research conduct and authorship and not only relegate them to middle-author positions by default [8]. Third, where feasible, cite local journals and local authors after careful literature search to enhance their visibility and impact [8]. Fourth, invest in mentorship and transfer of data, analyses, and technologies in low-resource settings [14]. Fifth, create opportunities for students and early-stage career researchers to participate in scholarly writing [9,12]. The power to write is invaluable in science because it determines a scientist’s productivity (i.e., output or number of authored papers) and impact (i.e., number of citations they accrue). While these 2 indicators of productivity and impact are the Holy Grail of academic success, it is important to question their appropriateness for use as success metrics in the scientific community when inequities exist in authorship [6,9,15].

## What can medical journals do to end parachute science?

For research to be validated it needs to be peer-reviewed and published, thus journals have the unique responsibility to increase awareness of—and eliminate—extractive research practices. Due to mounting recognition of the central role played by journals to ensure equity in scholarly publishing, the Royal Society of Chemistry has initiated collective action, the Joint Commitment for Action on Inclusion and Diversity in Publishing (https://www.rsc.org/new-perspectives/talent/joint-commitment-for-action-inclusion-and-diversity-in-publishing/). In 2021, *PLOS* announced a landmark policy on parachute science and inclusivity in global research. Foreign researchers are required to complete an “Inclusivity in global health questionnaire”—which aims to improve transparency in reporting of research performed outside researcher’s own country [16]. Beyond *PLOS*, other journals and publishers have committed to promoting fairness, equity, inclusivity, and integrity in collaborative research. For example, *Cell Press* instituted an “inclusion and diversity form” and *The Lancet* “will continue to reject papers with data from Africa that fail to acknowledge African collaborators” [17,18].

While these achievements signal progress toward scholarly inclusion and equity, they still do not address the glaring gap in equitable authorship—specifically, who holds first or last author positions [8]. Journals can safeguard justice in global research by instilling a culture of accountability in authorship assignment; however, this calls for reconsideration of how global research authorship is structured in the academic and scholarly publishing ecosystems. This is an exhortation to all journals to develop ad hoc policies to tackle this inequity in global research. Publications from research carried out in foreign contexts should have local researchers as either first or last authors. As part of *PLOS Medicine*’s commitment to address parachute science, we now require that local researchers be first or last authors of publications based on global research. With this initiative, we hope to correct long-debated academic power asymmetries that have lurked under hierarchies in the author by-line [6].
